# Premature Adult Death in Individuals Born Preterm: A Sibling Comparison in a Prospective Nationwide Follow-Up Study

**DOI:** 10.1371/journal.pone.0165051

**Published:** 2016-11-07

**Authors:** Kari R. Risnes, Kristine Pape, Johan H. Bjørngaard, Dag Moster, Michael B. Bracken, Pal R. Romundstad

**Affiliations:** 1 Department of Pediatrics, St Olav Hospital, University Hospital, Trondheim, Norway; 2 Institute of Public Health and General Practice, NTNU, Norwegian University of Science and Technology, Trondheim, Norway; 3 Forensic Department and Research Centre Bröset St. Olav's University Hospital, Trondheim, Norway; 4 Department of Global Public Health and Primary Care, University of Bergen, Bergen, Norway; 5 Department of Pediatrics, Haukeland University Hospital, Bergen, Norway; 6 Norwegian Institute of Public Health, Oslo, Norway; 7 Schools of Public Health and Medicine, Yale University, New Haven, Connecticut, United States of America; Institute of Preventive Medicine, DENMARK

## Abstract

**Background:**

Close to one in ten individuals worldwide is born preterm, and it is important to understand patterns of long-term health and mortality in this group. This study assesses the relationship between gestational age at birth and early adult mortality both in a nationwide population and within sibships. The study adds to existing knowledge by addressing selected causes of death and by assessing the role of genetic and environmental factors shared by siblings.

**Methods:**

Study population was all Norwegian men and women born from 1967 to 1997 followed using nation-wide registry linkage for mortality through 2011 when they were between 15 and 45 years of age. Analyses were performed within maternal sibships to reduce variation in unobserved genetic and environmental factors shared by siblings. Specific outcomes were all-cause mortality and mortality from cardiovascular diseases, cancer and external causes including accidents, suicides and drug abuse/overdoses.

**Results:**

Compared with a sibling born in week 37–41, preterm siblings born before 34 weeks gestation had 50% increased mortality from all causes (adjusted Hazard Ratio (aHR) 1.54, 95% confidence interval (CI) 1.17, 2.03). The corresponding estimate for the entire population was 1.27 (95% CI 1.09, 1.47). The majority of deaths (65%) were from external causes and the corresponding risk estimates for these deaths were 1.52 (95% CI 1.08, 2.14) in the sibships and 1.20 (95% CI 1.01, 1.43) in the population.

**Conclusion:**

Preterm birth before week 34 was associated with increased mortality between 15 and 45 years of age. The results suggest that increased premature adult mortality in this group is related to external causes of death and that the increased risks are unlikely to be explained by factors shared by siblings.

## Background

Modern neonatal intensive care and prenatal maternal corticosteroids were introduced in the late 1960s and considerably improved neonatal survival of preterm infants [1, 2]. Survivors of early neonatal intensive care are now reaching middle age and may be at risk for chronic disease and premature death.

Preterm birth has been consistently associated with reduced long-term cognitive function, as well as poor psychiatric[3, 4] and social well-being [2, 5]. Studies have reported adverse cardiovascular risk patterns in individuals born preterm, but increased cardiovascular disease has not been well documented[6–9]. Higher cancer risk after preterm birth has been indicated in some studies[10, 11] and preterm birth has been associated with increased all-cause mortality in young adulthood in a large Swedish population based study [12]. A weakness with many studies assessing long-term outcomes related to preterm birth has been a lack of data that can disentangle possible confounding by maternal and socioeconomic factors related to both risk of preterm birth and unfavourable adult social and health-related outcomes[2, 6, 12].

The main objective of this study was to study the association between preterm gestational age at birth and all-cause and cause-specific mortality in young adulthood, specifically mortality from external causes, cardiovascular diseases and cancer. We address potential residual confounding by comparing associations in the population cohort with associations within maternal sibships. We hypothesized that the associations between gestational age and mortality would remain when assessed within sibships, to indicate that genetic and environmental factors shared by siblings are unlikely to explain observed associations.

## Methods

### Study cohorts

Norwegian men and women born between 1967 and 1997 were followed for all-cause and cause specific mortality through 2011 when they were between 15 and 45 years of age. Only individuals with information on gestational age, with a gestational age recorded at 23 weeks through 44 weeks, and who were alive and residing in Norway at the age of 15 years were eligible. Eligible individuals were identified through the Medical Birth Registry of Norway (MBRN) which comprises compulsory registration of all births in the country from 1967.

Linkage to the Cause of Death Registry at Statistics Norway (CDRN), was ascertained by the 11-digit identification number allocated to all Norwegian residents. Linkage between siblings with the same mother was performed using the mother`s identification number. We identified two cohorts for analysis: 1) the population cohort consisted of the population as a whole, and 2) a sibling cohort consisting of all individuals identified in a family having at least two maternal siblings at least one of whom having died during follow-up.

### Variables

The exposure of interest was completed weeks of gestation according to the first day of the mother`s last menstrual period, as in previous publications from the cohort[2, 13]. Gestational age was assessed by a continuous measure (completed weeks) and grouped as: very preterm (23 to 33 weeks + 6 days), late preterm (34 to 36 weeks + 6 days), term (37 to 41 weeks + 6 days) and post- term (42 to 44 weeks + 6 days). An extremely preterm group (23 to 27 weeks + 6 days) was further identified. The choice of gestational age <34 weeks as a main exposure category was based on several considerations: infants born <34 weeks share factors related to biologic vulnerability including susceptibility to infections and preterm lung disease. Moreover, this group particularly benefitted from antenatal corticosteroids introduced in the early 70s[14] that substantially reduced neonatal mortality[2, 15]. Survival of extreme preterms (<28 weeks) was very low until the late 70s[2] and the present study included too few individuals in the extreme preterm group to reach robust conclusions. Information about the mother at time of birth was abstracted from MBRN and categorized: maternal age (<24, 25–29, 30–35, >35 years), parity (0, 1, 2,> = 3), length of education at child’s birth (0–2, 3–5, 6–8 years of education after high school). Offspring’s sex, multiple versus singleton birth and birth cohort (1967–1976, 1977–1986, and 1987–1997) were evaluated. Gestational age and sex-specific birth weight SD-scores were calculated as an indicator of gestational age related birth weight and as a proxy indicator of intrauterine growth. SD scores were created by subtracting the reference mean value of birth weight within each sex and gestational week stratum from the observed value [16], divided by the stratum specific standard deviation. Individuals with birth weight more than 6 SD below or above the mean birth weight for gestational age were excluded from the analyses.

Information on congenital malformations in the offspring was abstracted from the MBRN, and classified according to International Classification of Diseases, Eight and Tenth Editions (ICD8and 10) as 740–759 (ICD8) and Q00-99(ICD10).

### Mortality Outcomes in Adults

Individuals alive and living in the country by 15 years of age were individually linked to mortality and emigration information from the CDRN. Cause of death is determined by the doctor who examined the individual after death usually supported by previous patient history or autopsy. To ensure completeness, CDRN is cross linked to updated vital status recorded by the National Population Register. Follow-up for mortality started January 1981 at a minimum of 15 years of age and ended December 31^st^ 2011, at a maximum of 45 years of age. Cause of death was classified according to the International Classification of Disease (ICD), 8^th^ through 10^th^ editions: External Causes includes accidents/violence, suicide and substance-abuse/overdose (ICD 8/9: E800-999, ICD 10: V01-Y98), Cancer (including all neoplasms, 140–239, C00-D48) and Diseases of the Circulatory System (CVD, 390–459, I00-I99). Specific external causes of death studied were: accidents excluding poisonings (E800-E929, V01-X39, X50-59), suicide/intentional self-harm (E950-E959, X60-X84 Y87.0), accidental poisoning/substance abuse/overdose (303–305, F10-F19 X40-49). The study was approved by the Regional Committee for Medical Research Ethics in Southern and Eastern Norway (2012/188).

### Statistical methods

We used the Cox proportional hazards model to calculate hazard ratios (HR) for all cause and cause specific mortality according to gestational age. The proportional hazards assumption was tested graphically and by the use of Schoenfeld residuals. Basic models were age-adjusted using attained age as the time variable. Adjusted models included maternal age, parity, length of education, offspring sex, plurality and birth cohort. Potential confounding by intrauterine growth was addressed by adjusting for the SD score of birth weight for gestational age in a separate model and by sensitivity analyses excluding individuals with an SD score < three SD. Possible effects of congenital malformations or multiple pregnancy were evaluated by excluding individuals with these conditions in separate sensitivity analyses. In supplementary analyses, we explored a possible dose-response effect of the degree of preterm birth by further categorization of the <34 preterm group into extremely preterm (23 to 27 weeks + 6days) and very preterm (28 to 33 weeks + 6days). We evaluated potential effect modification owed to sex and birth order by including a product term between gestational age and sex/birth order, tested by a likelihood ratio test. Estimates precision was assessed by 95% confidence intervals (CI). In regression models, with weeks of gestation as a continuous variable, we calculated two-sided *P-*values from trend tests for the linear effects of gestational age; non-linear associations were tested by adding a quadratic term in the regression model and using a likelihood ratio test. To account for non-linear effects, we used restricted cubic splines with three knots in the graphical presentations of the associations. For the graphical presentations the estimates for predictions were calculated relative to gestational age 40 weeks (reference value with HR = 1.00). The sibling analyses estimated associations within maternal sibships in stratified cox models with mother’s identification number defining each stratum. Analyses were adjusted for maternal age, parity, offspring sex, plurality and birth cohort. These analyses use information only from sibships discordant for outcome status (death) and exposure (gestational age). Sibling analyses take advantage of the fact that siblings share many background factors that could potentially confound the associations between gestational age and mortality. Performing analyses within sibships is one approach to handling residual confounding from unobserved family-level confounders, including shared genetic and environmental factors[17] [18].

All statistical analyses and graphs were conducted using Stata for Windows (Version 12/13 StataCorp LP, 1985–2007).

## Results

### Cohort analysis

Among 1,727,494 individuals born in Norway from 1967 through 1996, 22,004 died before their 15^th^ birthday and another 15,841 were not registered as residents of Norway at the beginning of follow-up at 15 years of age. After excluding individuals with missing information 1,562,647 were included in the population cohort (S1 Fig). Among 1,245,318 individuals (80%) registered with at least one maternal sibling, we identified 29,536 individuals who belonged to one of 11,316 maternal sibling groups in which at least one sibling died. Characteristics of the population and sibling cohorts are presented in Table 1. The sibling cohort included a larger proportion of males and a larger proportion of participants born in the earliest birth cohorts compared to the population cohort.

**Table 1:** Baseline characteristics of a nationwide population cohort and a sibling based study born in Norway 1967–1997.

	Population ^a^, N = 1,562,647				Siblings ^b^, N = 29,536			
Categories of gestation	23–33 w+6d	34-36w+6d	37–41 w+6d	> = 42 w+0d	23–33 w+6d	34-36w+6d	37–41 w+6d	> = 42 w+0d
Number of participants (%)	19,597 (1)	61,082 (4)	1,265,248 (81)	216,720 (14)	350 (1)	1,249 (4)	23,676 (81)	4,261 (14)
Length of gestation, weeks (SD)	31(2)	35 (1)	40 (1)	42 (1)	31 (2)	35 (1)	40 (1)	42 (1)
Birth weight, g (SD)	1,850 (689)	2,740 (592)	3,540 (496)	3,720 (486)	2,012 (730)	2,773 (609)	3,520 (506)	3688 (501)
Female sex (%)	8,914 (45)	27,324 (45)	614,877 (49)	109,812 (51)	137 (39)	508 (37)	9,616 (41)	1,769 (42)
Non-singleton births (%)	2,335 (12)	9,302 (15)	20,659 (2)	774 (0)	39 (11)	198 (16)	575 (2)	23 (0)
Born 1967–76 (%)	6,097 (1)	21,709 (4)	469,814 (81)	82,810 (14)	172 (1)	700 (4)	13,529 (80)	2,476 (15)
Born 1977–86 (%)	5,242 (1)	16,735 (4)	375,259 (81)	64,784 (14)	115 (1)	401 (4)	7,474 (81)	1,345 (14)
Born 1987–97 (%)	8,258 (2)	22,638 (4)	420,175 (81)	69,126 (13)	63 (1)	148 (5)	2,673 (81)	440 (13)
Maternal age, no (%)								
<24y	7,460 (38)	23,519 (39)	460,333 (36)	93,249 (43)	153 (44)	550 (44)	10,118 (43)	2,058 (48)
25-29y	6,109 (31)	19,231 (31)	444,158 (35)	74,528 (34)	84 (24)	361 (29)	7,504 (32)	1,330 (31)
30-35y	3,973 (20)	11,893 (20)	252,927 (20)	36,149 (17)	79 (22)	229 (18)	4,162 (17)	640 (15)
≥35y	2,055 (11)	6,439 (10)	107,831 (9)	12,794 (6)	34 (10)	109 (9)	1,892 (8)	233 (5)
Maternal education, no (%)								
0-2y	13,164 (67)	39,276 (64)	751,920 (59)	133,641 (62)	218 (62)	729 (58)	12,437 (52)	2,343 (55)
3–5 y	5,612 (29)	18,753 (31)	437,662 (35)	72,395 (33)	118 (34)	497 (37)	9,658 (41)	1,675 (39)
> = 6 y	821 (4)	3,053 (5)	75,517 (6)	10,672 (5)	14 (4)	73 (5)	1,581 (7)	243 (6)
Maternal parity, no (%)								
0	9,749 (50)	28,437 (47)	508,437 (40)	101,638 (47)	118 (34)	397 (32)	7,093 (30)	1,561 (37)
1	5,454 (28)	18,079 (30)	446,635 (35)	69,789 (32)	106 (30)	390 (31)	8,280 (35)	1,373 (32)
2	2,794 (14)	9,228 (15)	209,896 (17)	31,336 (15)	69 (20)	266 (21)	5,052 (21)	814 (19)
> = 3	1,600 (8)	5,338 (9)	100,131 (8)	13,945 (6)	57 (17)	196 (16)	3,251 (14)	513 (12)

^a^ Norwegians born 1967–1997 who were living in the country and alive at 15 years of age and could be followed for mortality

^b^ Maternal siblings belonging to a sibling group in which at least one member died during follow-up

Percentages may not sum to 100 after rounding to nearest whole number

### Mortality

**Fig 1:**
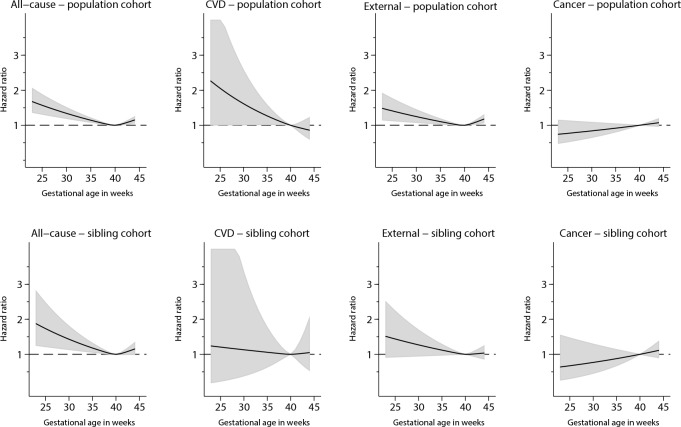
All-cause mortality and main categories of mortality risks (adjusted Hazard Ratios with 95% Confidence Intervals) according to gestational age at birth (completed weeks), in population (upper row) and sibling cohorts (lower row). Nation-wide cohort born in Norway 1967–1997.

At follow-up 14,919 individuals had died (11,570 in the sibling cohort) and nearly two thirds were from External Causes. Associations for all-cause mortality and for mortality from three main causes of death (External Causes, Cancer and CVD) are shown in Table 2 and illustrated in Fig 1. All-cause mortality was increased in individuals born before week 34 in the population cohort (adjusted Hazard Ratio (aHR) 1.27, 95% confidence interval (CI) 1.09, 1.47). Findings in the sibling cohort were similar. Thus, compared with a sibling born at term, preterm siblings born in week 23 through 33 had 54% increased risk of mortality from all causes (aHR 1.54 95% CI 1.17, 2.03). There was not strong evidence for increased mortality for the late preterm group (week 34 through 36).

**Table 2:** Mortality and gestational age by cause of death. Nation-wide cohort born in Norway 1967–1997.

Cause of death^e^ / Length of gestation Weeks (w)+days (d)	Population ^a^N = 1,562,647				Siblings ^b^N = 29,536			
	Deaths (N)	HR ^c^	95% CI ^c^	P trend	Deaths (N)	HR ^b^	95% CI ^b^	P trend
All causes of death								
22–33 w+6d	213	1.27	1.09, 1.47		158	1.54	1.17, 2.03	
34–36 w+6d	635	1.11	1.02, 1.20		488	1.00	0.86, 1.16	
37–41 w+6d	11,866	Ref			9,187	Ref		
> = 42 w+0d	2,205	1.08	1.03, 1.13		1,737	1.08	1.00, 1.18	
P trend-linear ^d^ /quadratic ^e^	14,919			0.04/<0.001	11,570			0.2/0.001
External causes of death ^f^								
22–33 w+6d	135	1.20	1.01, 1.43		98	1.52	1.08, 2.14	
34–36 w+6d	417	1.09	0.99, 1.21		320	0.94	0.78, 1.13	
37–41 w+6d	7,793	Ref			6,119	Ref		
> = 42 w+0d	1,456	1.08	1.03, 1.14		1,154	1.03	0.93, 1.15	
P trend-linear ^e^ /quadratic ^e^	9,744			0.7/<0.001	7,691			0.2/0.07
Cancer ^f^								
22–33 w+6d	25	1.30	0.97, 1.93		22	1.52	0.64, 3.67	
34–36 w+6d	58	0.86	0.66, 1.13		43	0.77	0.47, 1.27	
37–41 w+6d	1,472	Ref			1,113	Ref		
> = 42 w+0d	282	1.12	0.98, 1.27		217	1.13	0.88, 1.43	
P trend-linear ^e^ /quadratic ^e^	1,837			0.2/0.4	1,373			0.3/0.2
Cardiovascular Diseases ^f^								
22–33 w+6d	15	1.58	0.94, 2.64		8	1.01	0.29, 3.55	
33–36 w+6d	39	1.18	0.85, 1.64		29	1.31	0.68, 2.52	
37–41 w+6d	674	Ref			499	Ref		
> = 42 w+0d	105	0.90	0.73, 1.11		79	1.01	0.70, 1.46	
P trend-linear ^e^ /quadratic ^e^	833			0.01/1.01	615			0.8/0.9

HR: Hazard ratio, CI: Confidence Interval

^a^ Norwegians born 1967–1997 who were living in the country and alive at 15 years of age and could be followed for mortality

^b^ Maternal siblings belonging to a sibling group in which at least one member died during follow-up

^c^ Adjusted for sex, birth cohort (1967–1976, 1977–1986, and 1987–1997), maternal age (<24, 25–29, 30–35, ≥35 years), maternal parity (0,1, 2, ≥3), maternal education (0–2, 3–5, 6–8 years of education after high school), singleton born (y/n)

^d^ P linear: value for linear association across weeks of completed gestational age.

^e^ P quadratic: non-linear associations, tested by using a likelihood ratio test, by adding a quadratic term in the regression model.

^f^ Cause of death (ICD10 and ICD9 codes): External causes (V01-Y89 and E800-E999, included drug/alcohol-related deaths F10-19, 303–305); Cancer (C00-D48, 140–239); Cardiovascular Diseases (I00-I99, 390–459)

The group born before 34 weeks’ gestation was at increased risk of death from External Causes in the population cohort (aHR 1.20, 95% CI 1.01, 1.43) and in the sibling cohort (aHR 1.52, 95% CI 1.08, 2.14). For death from neoplasms, there were few cases to yield precise estimates, although the effect estimates for the most preterm group were comparable to those for all-cause mortality. Mortality from CVD accounted for only 6% of all deaths and the analyses included a relatively low number of cases. We observed an inverse linear association between gestational age and CVD mortality in the population cohort, but these findings were not supported in the sibling cohort.

Associations between gestational age and specific groups of external causes (accidents/violence, suicide, and substance abuse/ overdose) are shown in Table 3, and although some results lack power for robust conclusions, they indicate that shorter gestational age is associated with higher mortality from these three causes of death. In the sibling cohort, we found a two-fold increased risk of death due to accidents and violence for gestational age less than 34 weeks compared to term births. The corresponding result for suicide was similar, but a lower number of cases yielded a less precise estimate. The estimates for substance abuse related deaths were attenuated in the sibling analyses and do not support any association.

**Table 3:** Mortality and gestational age by external cause of death. Nation-wide cohort born in Norway 1967–1997.

Cause of death / Length of gestation Weeks (w)+days (d)	Population ^a^N = 1,562,647				Siblings ^b^N = 29,536			
	Deaths (N)	HR ^c^	95% CI ^c^	P trend	Deaths (N)	HR ^c^	95% CI ^c^	P trend
Accidents and violence ^f^								
22–33 w+6d	56	1.20	0.92, 1.57		43	1.87	1.12, 3.10	
34–36 w+6d	161	1.02	0.87, 1.19		146	1.03	0.77, 1.38	
37–41 w+6d	3,204	Ref			2,538	Ref		
= 42 w+0d	554	1.00	0.92, 1.10		443	0.90	0.77, 1.07	
P trend-linear ^d^ /quadratic ^e^	3,975			0.7/0.08	3,154			0.007/0.04
Suicide ^f^								
22–33 w+6d	42	1.16	0.85, 1.57		28	1.96	0.97, 3.98	
34–36 w+6d	142	1.14	0.96, 1.35		112	1.08	0.78, 1.49	
37–41 w+6d	2,587	Ref			2,054	Ref		
> = 42 w+0d	503	1.14	1.04, 1.26		397	1.05	0.88, 1.26	
P trend-linear ^d^ /quadratic ^e^	3,274			1.0/ 0.006	2,591			0.5/0.002
Substance abuse/overdose ^f^								
22–33 w+6d	31	1.22	0.87, 1.75		24	1.07	0.55, 2.01	
34–36 w+6d	104	1.21	0.99, 1.48		69	0.65	0.44, 0.97	
37–41 w+6d	1,745	Ref			1,322	Ref		
> = 42 w+0d	347	1.13	1.00, 1.26		278	1.15	0.92, 1.44	
P trend-linear ^d^ /quadratic ^e^	2,227			0.6/0.3	1,693			0.3/ 0.6

HR: Hazard ratio, CI: Confidence Interval

^a^ Norwegians born 1967–1997 who were living in the country and alive at 15 years of age and could be followed for mortality

^b^ Maternal siblings belonging to a sibling group in which at least one member died during follow-up

^c^ Adjusted for sex, birth cohort (1967–1976, 1977–1986, and 1987–1997), maternal age (<24, 25–29, 30–35, ≥35 years), maternal parity (0,1, 2, ≥3), maternal education (0–2, 3–5, 6–8 years of education after high school), singleton born (y/n)

^d^ P linear: value for linear association across weeks of completed gestational age

^e^ P quadratic: non-linear associations, tested by using a likelihood ratio test, by adding a quadratic term in the regression model

^f^ Cause of death (ICD10 and ICD9 codes): Accidents and violence (V01-X39, X50-59, Y85-86, E800-E929), Suicide (X60-X84, Y87.0, E950-E959), Substance abuse/overdoses (F10-F19 X40-49, 303–305)

### Supplementary analyses

Exclusion of individuals born after a multiple pregnancy, with a neonatal diagnosis of congenital malformations, a SD score for birthweight higher than three, or adjustment for birth weight for gestational age (data not shown), did not meaningfully alter the estimates. Since we found limited evidence for effect modification by sex (p-value 0.3), we performed combined analyses of men and women, and included sex as a co-variable in the regression analyses. There was weak evidence of effect modification by birth order (p-value 0.1). An observed tendency of non-proportional hazards for gestational age and mortality was accounted for by performing separate analyses stratified by age during follow-up (Table 4). There was some evidence that risk estimates for overall mortality and mortality from external causes increased by increasing age at death. The adjusted HR for all-cause mortality after 35 years of age for the group with gestational age 33 weeks or less, compared to those born at term, was 1.60 (95%CI 1.18, 2.17) in the population cohort. The corresponding estimate for External Causes of death was 2.22 (95% CI 1.50, 3.29). Supplementary analyses indicated a dose-response relationship with higher estimates for the lowest category (<28 weeks) (Table A in S1 File and Table B in S1 File), but these analyses lack power for any robust conclusions.

**Table 4:** Mortality and gestational age by cause of death. Nation-wide cohort born in Norway 1967–1997. Analyses stratified by age at death.

	Age 15–25		Age 25–35		Age 35–45	
Cause of death^c^	HR ^a^ population	HR ^a^ siblings ^b^	HR ^a^ population	HR ^a^ sibling ^b^	HR ^a^ population	HR ^a^ siblings ^b^
All causes						
22–33 weeks+6days	1.13 (0.91, 1.39)	1.45 (0.99, 2.13)	1.32 (1.05, 1.65)	1.58 (1.01, 2.45)	1.60 (1.18, 2.17)	1.99 (0.81, 4.93)
34–36 weeks+6days	1.08 (0.95, 1.21)	1.02 (0.83, 1.25)	1.05 (0.92, 1.21)	0.99 (0.77, 1.28)	1.32 (1.10, 1.58)	0.94 (0.62, 1.43)
37–41 weeks+6days						
> = 42 weeks	1.09 (1.02, 1.17)	1.06 (0.95, 1.19)	1.10 (1.02, 1.19)	1.16 (1.01, 1.33)	1.00 (0.89, 1.12)	0.99 (0.75, 1.29)
Cancer						
22–33 weeks+6days	0.98 (0.44, 2.21)	2.74 (0.58, 12.90)	1.55 (0.85, 2.83)	1.35 (0.40, 4.59)	1.33 (0.66, 2.45)	0.46 (0.04, 5.76)
34–36 weeks+6days	1.12 (0.73, 1.70)	1.37 (0.61, 3.05)	0.69 (0.42, 1.12)	0.40 (0.17, 0.93)	0.83 (0.52, 1.33)	1.04 (0.32, 3.40)
37–41 weeks+6days						
> = 42 weeks	1.01 (0.80, 1.29)	1.03 (0.69, 1.52)	1.24 (1.01, 1.52)	1.22 (0.82, 1.81)	1.08 (0.86, 1.35)	1.15 (0.68, 1.94)
External Causes						
22–33 weeks+6days	1.07 (0.83, 1.37)	1.29 (0.81, 2.03)	1.11 (0.82, 1.49)	1.51 (0.86, 2.67)	2.22 (1.50, 3.29)	5.61 (1.40, 22.49)
34–36 weeks+6days	1.01 (0.88, 1.17)	0.91 (0.71, 1.16)	1.04 (0.88, 1.22)	1.08 (0.78, 1.50)	1.66 (1.29, 2.12)	0.75 (0.41, 1.34)
37–41 weeks+6days						
> = 42 weeks	1.11 (1.02, 1.20)	1.03 (0.90, 1.18)	1.06 (0.97, 1.16)	1.06 (0.89, 1.27)	1.08 (0.91, 1.29)	0.92 (0.59, 1.45)
Cardiovascular (833)						
22–33 weeks+6days	0.37 (0.05, 2.66)		2.93 (1.50, 5.72)	2.47 (0.46, 13.15)	1.31 (0.54, 3.18)	3.10 (0.14, 70.32)
34–36 weeks+6days	1.05 (0.53, 2.07)	1.34 (0.27, 6.62)	1.56 (0.95, 2.56)	1.39 (0.49, 3.93)	0.95 (0.54, 1.66)	1.30 (0.33, 5.08)
37–41 weeks+6days						
> = 42 weeks	0.90 (0.59, 1.36)	0.95 (0.51, 1.77)	1.06 (0.76, 1.46)	1.56 (0.86, 2.84)	0.77 (0.54, 1.09)	0.65 (0.28, 1.52)

HR: Hazard ratio, CI: Confidence Interval

^a^ Maternal siblings belonging to a sibling group in which at least one member died during follow-up

^b^ Adjusted for sex, birth cohort (3), maternal age (<24, 25–29, 30–35, ≥35 years), maternal parity (0,1, 2, ≥3), maternal education (0–2, 3–5, 6–8 years of education after high school), singleton (y/n)

^c^ Cause of death (ICD10 and ICD9 codes): Accidents and violence (V01-X39, X50-59, Y85-86, E800-E929), Suicide (X60-X84, Y87.0, E950-E959), Substance abuse/overdoses (F10-F19 X40-49, 303–305)

## Discussion

In this complete nation-wide follow-up of 1.5 million adults, shorter length of gestation was associated with higher mortality in early adulthood and the risk estimate was particularly high for external causes of deaths after 35 years of age. The main findings were robust in sibship analyses that control for environmental and genetic factors shared between siblings with same mother.

### Comparison with other studies

#### Mortality

Many historical cohort studies have reported that lower birth weight is associated with increased adult mortality[19]. However, birth weight is strongly related to both gestational age at birth, as well as maternal genetic and socioeconomic factors that may affect intrauterine growth. The separate roles of gestational age and family factors were only partly disentangled in previous mortality studies[19]. However, a recent sibling analyses on Swedish data concluded that shared family factors could not explain increased mortality in individuals born with lower birth weights [20]. A recent study assessed mortality related to small for gestational age status and concluded that individuals born SGA were at increased mortality risk in childhood, but not after 30 years of age[21]. Thus, these two studies may support a conclusion that neither family factors nor intrauterine growth restriction can explain increased long-term mortality in individuals born small.

Some recent studies [13, 18, 22] report an increased long-term mortality risk related to preterm birth, but in less detail on mortality causes and with shorter follow-up than the present study.

Using data from The Swedish Medical Birth Registry, Crump and co-workers [22] showed that increased overall mortality related to preterm birth was strong in childhood, tended to disappear in adolescents (13–17 years) and reappeared in young adulthood (18–36 years). The same Swedish data was recently re-analysed[18], and the sibling analyses showed no attenuation of the inverse association between gestational age and overall mortality between 1 and 36 years of age. Although childhood mortality was the main contributor to overall mortality in that study, the conclusion supports our finding that the association between gestational age and long-term all-cause mortality is robust to confounders shared by siblings.

#### Mental illness, social function and cognition

Susceptibility to mental illness, including attention problems and factors related to cognitive skills and social well-being, could possibly explain some of the observed increased risk of deaths from external causes of death in the present study. Large Scandinavian register-based data analyses have found associations between preterm birth and a wide range of educational, social, cognitive and mental health outcomes in adolescence and young adulthood [2–4, 18, 23–27]. However, a causal relationship between preterm birth and cognition was recently questioned in a Swedish analysis which showed that the effect of preterm birth (<30 weeks) on low school grades attenuated in sibling analyses[28].

Overall, outcomes related to mental and social well-being have been well described in adolescents born preterm, but is less well documented in adults. Some studies suggest increasing risk of psychopathology and cognitive problems as children born preterm reach middle-age [29–31], supporting our findings of particularly high risk in the 35–45 year group. In the present analyses, we observed a tendency of increased suicide risk in individuals born preterm and a causal relationship may be supported by the observed high risk estimates for suicide in the preterm group in the sibling comparison. In contrast to this finding, sibling analyses on preterm birth and suicide attempts in Swedish data did not support a causal relationship.[18]. An explanation for this discrepancy might be that our analyses included only completed suicides and other mechanisms may apply to suicide attempts.

#### Cardiovascular risk and disease

Our results do not support a robust association between preterm gestational age and early adult cardiovascular mortality, although increased risk estimates for CVD death was observed for deaths after 20 years of age. However, firm conclusions are hampered by low number of cardiovascular deaths, possibly explained by the still relatively young age of this cohort. Our findings of a possible attenuation of risk estimates for CVD deaths in the sibling study compared to in the population cohort could indicate that increased CVD risk after preterm birth is confounded by family factors. This interpretation may be supported by a Swedish follow-up study[32] that reported that mothers who had previously given preterm birth themselves had increased CVD mortality later in life, suggestive of genetic factors linked to preterm labour and later CVD. Cardiovascular and metabolic long-term outcomes after preterm birth have been extensively studied and debated. In a recent meta-analysis [8] the authors concluded that preterm birth was not associated with unfavourable metabolic outcomes in adulthood (mean 20–30 years) although the analysis reported higher blood pressure in adults born preterm. In contrast, a Finnish follow-up study found preterm birth associated with higher fat percentage and risk of metabolic syndrome in young adults[33]. Previous studies did not indicate robust associations of preterm birth with ischemic heart disease in historic cohorts that were limited by low precision in gestational age assessments[34–37]. More recent cohorts support these findings[9, 38], although studies are limited by a low number of cases of ischemic heart disease at these relatively young adult ages. For adult cerebrovascular diseases, studies indicate an increased risk in adults born preterm[9, 34, 35]. In our study, there were too few cardiovascular deaths to robustly evaluate deaths due to specific cardiovascular diagnoses.

### Weaknesses and strengths

The present results were based on a large population based study with almost complete follow-up. De-identified information on exposure, outcome and cofactors were abstracted and linked by high quality Norwegian national registries (NMBR: https://www.fhi.no/en/op/data-access-from-health-registries-health-studies-and-biobanks/medical-birth-registry-and-registry-of-pregnancy-termination/core-articles and CDRN: https://www.fhi.no/en/hn/health-registries/cause-of-death-registry/)

Another important strength is that we could assess mortality within many sibships. We accounted for unobserved factors, shared between siblings, which could influence the risk of being born preterm and for premature mortality, such as educational and socioeconomic factors of the household and genetic variability. Although comparing differentially exposed siblings would take into account shared confounding, non-shared confounding and random measurement error can still bias the results of sibling analyses [39]. Furthermore, we cannot exclude the possibility that comparisons within families may be vulnerable to time-dependent factors that could influence preterm birth risk and the family environment. Differential effects on siblings (such as parental death or illness, divorce, unemployment, foster care, domestic violence) may have occurred at different stages of the siblings’ lives. The risk estimates from our analyses were typically higher in the sibling comparisons compared to the population sample. This could be due to the fact that the sibling comparisons may provide a better adjustment for rather subtle but potentially important factors for which information is usually not available. The within sibling analyses were restricted to sibships with discordant exposure, which might be vulnerable to selection bias and measurement error^20^. We would rather expect reduced estimates in case of non-differential measurement error, but we cannot rule out the possibility of selection bias from non-shared factors due to restriction on discordant exposure status. Sensitivity analyses limiting the sample to one-child families yielded similar estimates to those in families with more than one child and could therefore not explain the higher estimates in the sibling sample. Other studies have also shown higher magnitudes of estimates in sibling comparisons[20, 40].

The study included a high number of cases that makes chance an unlikely explanation for our main findings. However, the cohort is still relatively young and few have reached an age when death from chronic diseases is prevalent. It is a shortcoming that we cannot draw strong conclusions for more specific categories of cause-specific adult deaths. In the present study we could only include a relatively low number of individuals born extreme preterm (gestational age <28 weeks) and our results do not allow specific conclusions for this group. Neonatal treatment and survival for individuals born preterm differs by time and place of birth[2, 41], and long-term outcomes may therefore be dependent on these factors. Our data did not permit studying possible birth cohort effects on long-term mortality. A longer follow-up time is needed to study adult mortality for the increasing number of survivors after extreme preterm birth, born in the post-surfactant era. Preterm birth rates in Norway increased by 25% from 1980–98[42], but are still low at 6.1% for 2015 http://statistikkbank.fhi.no/mfr/ compared to many other parts of the developed world. Generalizability of long-term risk to other countries with higher prevalence of preterm births or different perinatal care may therefore be limited.

Specific causes of death, particularly the distinction between accidents and suicide, may be underreported. This would only bias our results if the underreporting was associated with gestational age at birth. The prospective design of the study makes such bias unlikely. It is a weakness that gestational age is assessed by last menstrual period and so may be more prone to misclassification than modern standards using ultrasound. However, sensitivity analyses that excluded extreme birth weights for gestational ages and analyses that adjusted for birth weight did not affect the results. Misclassification of gestational age is most likely to have led to a non-differential bias that would tend to attenuate associations.

### Relevance

Distribution of causes of death in adolescents and young adults in the present study corresponds well with global data showing that injuries and self-harm are the main contributors to loss of life years in this age group [43, 44]. Thus, increased mortality risk from external causes in individuals born preterm has public health interest. The present findings should be interpreted with caution. Recent cohorts of preterm infants have higher neonatal survival and are exposed to changing medical treatments that may cause different long-term risk profiles from cohorts born three to four decades ago. We only included early adult deaths, and changing patterns of morbidity and mortality by age may produce different results with longer follow-up. Nevertheless, the results warrant close attention to risk assessment in young adults born preterm and to the importance of following cohorts of individuals born preterm as they age.

## Supporting Information

S1 Fig Establishment of cohort. Nation-wide cohort born in Norway 1967–1997.
(TIF)

S1 File **Table A**. Mortality and gestational age by cause of death. Nation-wide cohort born in Norway 1967–1997. **Table B.** Mortality and gestational age by external cause of death Nation-wide cohort born in Norway 1967–1997.
(DOCX)

## Abbreviations

CVD: cardiovascular diseases
