# Supplementary figures and images for: Premature Adult Death in Individuals Born Preterm: A Sibling Comparison in a Prospective Nationwide Follow-Up Study
Source: PLoS One. 2016 Nov 7;11(11):e0165051. doi: 10.1371/journal.pone.0165051 (PMC5098830; doi:10.1371/journal.pone.0165051)

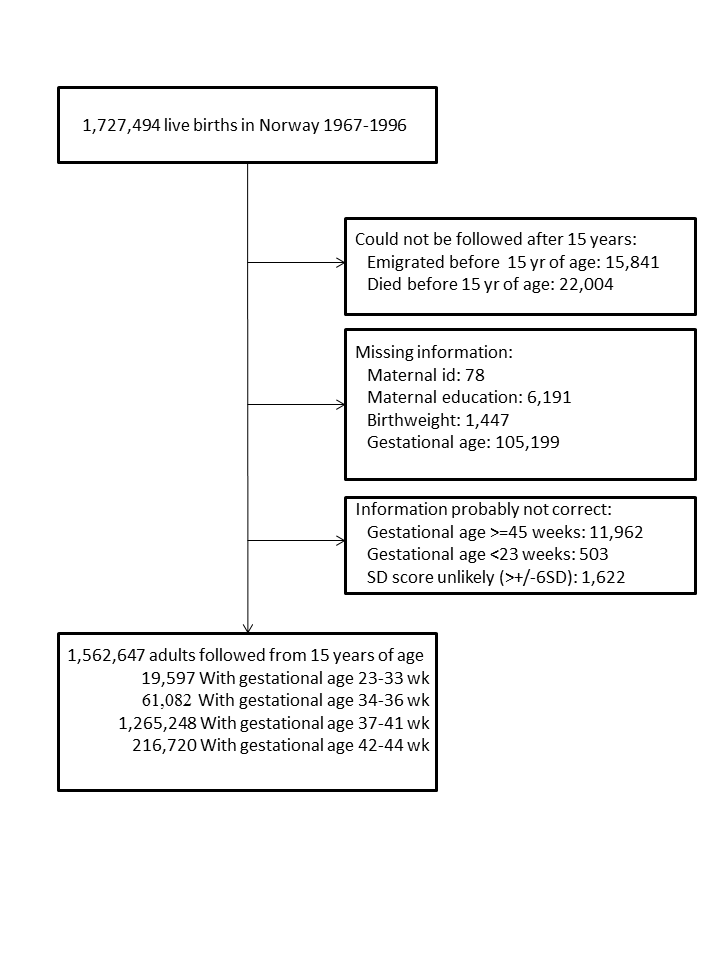

Supplement: S1 Fig — Nation-wide cohort born in Norway 1967–1997. (TIF) [file pone.0165051.s001.tif]
