# Supplementary material for: Premature Adult Death in Individuals Born Preterm: A Sibling Comparison in a Prospective Nationwide Follow-Up Study
Source: PLoS One. 2016 Nov 7;11(11):e0165051. doi: 10.1371/journal.pone.0165051 (PMC5098830; doi:10.1371/journal.pone.0165051)
Supplement: S1 File — Table A. Mortality and gestational age by cause of death. Nation-wide cohort born in Norway 1967–1997. Table B. Mortality and gestational age by external cause of death Nation-wide cohort born in Norway 1967–1997. (DOCX) [file pone.0165051.s002.docx]

**Supplemental Table A**. Mortality and gestational age by cause of death. Nation-wide cohort born in Norway 1967-1997.

| Cause of death^b^  Length of gestation  Weeks (w)+days (d) | Population cohort  N=1,562,647 | | |  | Sibling cohort^a^  N=29,536 | | |
| --- | --- | --- | --- | --- | --- | --- | --- |
|  | Deaths (N) | HR^c^ | 95% CI^a^ |  | Deaths (N) | HR^a^ | 95% CI^a^ |
|  |  |  |  |  |  |  |  |
| All causes of death | 14,919 |  |  |  | 11,570 |  |  |
| 23-27 w+6d | 12 | 1.6 | 0.9, 2.8 | | 8 | 2.0 | 0.6, 6.6  1.1, 2.0  0.9, 1.2 |
| 28-33 w+6d | 201 | 1.3 | 1.1, 1.4 | | 150 | 1.5 |  |
| 34-36 w+6d | 635 | 1.1 | 1.0, 1.2 | | 488 | 1.0 |  |
| 37-41 w+6d | 11,866 | Ref |  |  | 9,187 | Ref |  |
| >=42 w+0d | 2,205 | 1.1 | 1.0, 1.1 | | 1,737 | 1.1 | 1.0, 1.2 |
|  |  |  |  |  |  |  |  |
| External causes of death^b^ | 9,744 |  |  |  | 7,649 |  |  |
| 23-27 w+6d | 8 | 1.6 | 0.8, 3.1  0.9, 2.0  1.0, 1.2 | | 6 | 2.4 | 0.5, 10.3  1.0, 2.1  0.8, 1.1 |
| 28-33 w+6d | 127 | 1.2 |  |  | 92 | 1.5 |  |
| 34-36 w+6d | 417 | 1.1 |  |  | 320 | 0.9 |  |
| 37-41 w+6d | 7,793 | Ref |  |  | 6,119 | Ref |  |
| >=42 w+0d | 1,456 | 1.1 | 1.0, 1.1 | | 1,154 | 1.0 | 0.9, 1.1 |
|  |  |  |  |  |  |  |  |
| Cancer^b^ | 1,837 |  |  |  | 1,373 |  |  |
| 23-27 w+6d | 0 | - |  |  | 0 |  |  |
| 28-33 w+6d | 25 | 1.4 | 0.9, 2.0  0.7, 1.1 | | 22 | 1.5 | 0.6, 3.5  0.4, 1.2 |
| 34-36 w+6d | 58 | 0.9 |  |  | 43 | 0.7 |  |
| 37-41 w+6d | 1,472 | Ref |  |  | 1,113 | Ref |  |
| >=42 w+0d | 282 | 1.1 | 1.0, 1.3 | | 217 | 1.1 | 0.9, 1.4 |
|  |  |  |  |  |  |  |  |
| Cardiovascular Diseases | 833 |  |  |  | 615 |  |  |
| 23-27 w+6d | 0 | - |  |  | 0 |  |  |
| 28-33 w+6d | 15 | 1.6 | 1.0, 2.8  0.9, 1.6 | | 8 | 1.1 | 0.3, 3.9  0.7, 2.6 |
| 34-36 w+6d | 39 | 1.2 |  |  | 29 | 1.4 |  |
| 37-41 w+6d | 674 | Ref |  |  | 499 | Ref |  |
| >=42 w+0d | 105 | 0.9 | 07, 1.1 | | 79 | 1.0 | 0.7, 1.5 |

^a^ Maternal siblings belonging to a sibling group in which at least one member died during follow-up

^b^ Adjusted for sex, birth cohort (1967-1976, 1977-1986, and 1987-1997), maternal age (<24, 25-29, 30-35, ≥35 years), maternal parity (0,1, 2, ≥3), maternal education (0-2, 3-5, 6-8 years of education after high school), singleton born (y/n)

^c^ Cause of death (ICD10 and ICD9 codes): External causes (V01-Y89 and E800-E999, included drug/alcohol-related deaths F10-19, 303-305); Cancer (C00-D48, 140-239); Cardiovascular Diseases (I00-I99, 390-459)

.

**Supplemental Table B.** Mortality and gestational age by external cause of death Nation-wide cohort born in Norway 1967-1997.

| Cause of death^b^  Length of gestation  Weeks(w) +days(d) | Population cohort  N=1,562,647 | | |  | Sibling cohort^a^  N=29,536 | | |
| --- | --- | --- | --- | --- | --- | --- | --- |
|  | Deaths (N) | HR^c^ | 95% CI^a^ |  | Deaths (N) | HR^a^ | 95% CI^a^ |
|  |  |  |  |  |  |  |  |
| Accidents and violence^c^ | 3,975 |  |  |  | 3,154 |  |  |
| 23-27 w+6d | 5 | 2.3 | 1.0, 5.6 |  | 4 | 4.9 | 0.7, 33.6 |
| 28-33 w+6d | 32 | 1.2 | 0.8, 1.7 |  | 23 | 2.2 | 1.1, 4.2 |
| 34-36 w+6d | 180 | 1.0 | 0.9, 1.2 |  | 146 | 1.0 | 0.8, 1.4 |
| 37-41 w+6d | 3,204 | Ref |  |  | 2,538 | Ref |  |
| >=42 w+0d | 554 | 1.0 | 0.9, 1.1 |  | 443 | 0.9 | 0.8, 1.1 |
|  |  |  |  |  |  |  |  |
| Suicide^c^ | 3,274 |  |  |  | 2,591 |  |  |
| 23-27 w+6d | 2 | 1.2 | 0.3, 4.7 |  | 1 |  |  |
| 28-33 w+6d | 40 | 1.2 | 0.8, 1.6 |  | 27 | 1.9 | 0.9, 3.8 |
| 34-36 w+6d | 142 | 1.1 | 1.0, 1.4 |  | 112 | 1.1 | 0.8, 1.5 |
| 37-41 w+6d | 2,587 | Ref |  |  | 2,054 | Ref |  |
| >=42 w+0d | 503 | 1.2 | 1.0, 1.3 |  | 397 | 1.0 | 0.9, 1.2 |
|  |  |  |  |  |  |  |  |
| Substance abuse/overdose^c^ | 2,227 |  |  |  | 1,693 |  |  |
| 23-27 w+6d | 1 | 0.9 | 0.1, 6.4 |  | 1 |  |  |
| 28-33 w+6d | 30 | 1.2 | 0.9, 1.8 |  | 23 | 1.1 | 0.6, 2.2 |
| 34-36 w+6d | 104 | 1.2 | 1.0, 1.5 |  | 69 | 0.6 | 0.4, 1.0 |
| 37-41 w+6d | 1,745 | Ref |  |  | 1,322 | Ref |  |
| >=42 w+0d | 347 | 1.1 | 1.0, 1.3 |  | 278 | 1.2 | 0.9, 1.4 |

^a^ Maternal siblings belonging to a sibling group in which at least one member died during follow-up

^b^Adjusted for sex, birth cohort (1967-1976, 1977-1986, and 1987-1997), maternal age (<24, 25-29, 30-35, ≥35 years), maternal parity (0,1, 2, ≥3), maternal education (0-2, 3-5, 6-8 years of education after high school), singleton born (y/n)

^c^ Causes of death (ICD10 and ICD9 codes): Accidents and violence (V01-X39, X50-59, Y85-86, E800-E929), Suicide (X60-X84, Y87.0, E950-E959), Substance abuse/overdoses (F10-F19 X40-49, 303-305)

.
